# Assessment of behavioral, morphological and electrophysiological changes in prenatal and postnatal valproate induced rat models of autism spectrum disorder

**DOI:** 10.1038/s41598-021-02994-6

**Published:** 2021-12-06

**Authors:** Katarine Fereshetyan, Vergine Chavushyan, Margarita Danielyan, Konstantin Yenkoyan

**Affiliations:** 1 Neuroscience Laboratory, Cobrain Center, Yerevan State Medical University named after M. Heratsi, 2 Koryun Str., 0025 Yerevan, Armenia; 2 Department of Biochemistry, Yerevan State Medical University named after M. Heratsi, Yerevan, Armenia; 3 Laboratory of Neuroendocrine Relations, L. A. Orbeli Institute of Physiology NAS, Yerevan, Armenia; 4 Laboratory of Histochemistry and Electromicroscopy, L. A. Orbeli Institute of Physiology NAS, Yerevan, Armenia

**Keywords:** Neuroscience, Diseases

## Abstract

Autism spectrum disorders (ASD) are neurodevelopmental disorders, that are characterized by core symptoms, such as alterations of social communication and restrictive or repetitive behavior. The etiology and pathophysiology of disease is still unknown, however, there is a strong interaction between genetic and environmental factors. An intriguing point in autism research is identification the vulnerable time periods of brain development that lack compensatory homeostatic corrections. Valproic acid (VPA) is an antiepileptic drug with a pronounced teratogenic effect associated with a high risk of ASD, and its administration to rats during the gestation is used for autism modeling. It has been hypothesized that valproate induced damage and functional alterations of autism target structures may occur and evolve during early postnatal life. Here, we used prenatal and postnatal administrations of VPA to investigate the main behavioral features which are associated with autism spectrum disorders core symptoms were tested in early juvenile and adult rats. Neuroanatomical lesion of autism target structures and electrophysiological studies in specific neural circuits. Our results showed that prenatal and early postnatal administration of valproate led to the behavioral alterations that were similar to ASD. Postnatally treated group showed tendency to normalize in adulthood. We found pronounced structural changes in the brain target regions of prenatally VPA-treated groups, and an absence of abnormalities in postnatally VPA-treated groups, which confirmed the different severity of VPA across different stages of brain development. The results of this study clearly show time dependent effect of VPA on neurodevelopment, which might be explained by temporal differences of brain regions’ development process. Presumably, postnatal administration of valproate leads to the dysfunction of synaptic networks that is recovered during the lifespan, due to the brain plasticity and compensatory ability of circuit refinement. Therefore, investigations of compensatory homeostatic mechanisms activated after VPA administration and directed to eliminate the defects in postnatal brain, may elucidate strategies to improve the course of disease.

## Introduction

Autism spectrum disorders (ASD) are neurodevelopmental disabilities with unknown etiology, which characterized by two core symptoms: alterations of social communication and restrictive or repetitive behavior^1^. Despite decades of research the etiology and pathophysiology of disease is still unknown, however, there is a strong interaction between genetic and environmental factors^2^. One of the intriguing points in autism research among the others is identifying the vulnerable time periods of development deviations without compensatory homeostatic corrections. Generally, autism pathogenesis showed altered synaptogenesis and aberrant connectivity of networks mostly in high order brain regions, which are responsible for the social behavior and communication^3,4^. Notably, brain regions as prefrontal cortex and amygdala continue plastic changes even at postnatal period of development^5,6^. At this time period brain maturation continue, particularly pruning of synapses and refinement of neuronal connections^7^, gliogenesis^8,9^ occur. In this context it is important to pay attention on brain development differences in humans and rats. It is estimated that postnatal days 1–10 of rat development corresponds to the 3rd trimester in humans gestation according to the level of brain development^10^. The other critical change during the brain maturation is a switch of GABA excitatory effect to inhibitory^11,12^.

Among the environmental factors involved in ASD pathogenesis, it has been documented the teratogenic effect of antiepileptic drug valproic acid (VPA)^13^, which is approved for use in epilepsy and some psychiatric diseases including bipolar and mood disorders^14^. Exposure to valproic acid during the 1st trimester of gestation^15^ is highly associated with neurodevelopmental delay and significantly increased risk of autism spectrum disorders in offspring^16–18^. Based on the above valproate administration to pregnant rats on gestational 12.5 day becomes one of the validated animal model of autism^19–21^.One of the known effects of VPA is an elevation of GABA level^22,23^ and inhibition of histone deacetylase^24^. Increased level of GABA may alter excitatory and inhibitory neurotransmission. A tight balance between excitation and inhibition in synaptic inputs to a neuron and in neural circuits is important for normal brain development and function. Specific factors that contribute to synaptic excitatory/inhibitory (E/I) balance would include excitatory and inhibitory synapse development, synaptic transmission and plasticity, downstream signaling pathways, homeostatic synaptic plasticity, and intrinsic neuronal excitability. E/I imbalance has become a dominant theory on the pathogenesis of various autism spectrum disorders^25,26^. In sum, cellular E/I balance elevation is a key factor in pathophysiology of social and information processing dysfunction^26^. Depending on the location and severity of imbalance across the brain regions, as well as timing of critical periods, a spectrum of ASD phenotypes would result. Also, aberrant connectivity of numerous functional networks have significant role in ASD neuropathophysiological mechanisms^27–29^. Different aspects of the behavioral phenotype are likely to result from alterations in specific circuits. Social behavior is strongly regulated by PFC^30^, amygdala^31^ and there are strong associations between the cerebellum and the PFC, and abnormalities in both areas have been associated with the severity of ASD symptoms^32^. Many studies indicate that hyperactivity of prefrontal cortex lead to the some symptoms of autism such as sociability and attention deficits, multi-tasking and repetitive behaviors^33^.

Thus, it is attractive to hypothesize that valproic acid induced damage and functional alterations of autism target structures may occur and evolve during early postnatal life of rats^34–36^. To test this hypothesis behavioral, morphological and electrophysiological studies were carried out on animals exposed to valproic acid on prenatal and postnatal periods. Moreover, we aimed to assess the severity of VPA-induced changes in brain, estimate the manifestation and duration of symptoms, as well as possibility of their normalization during the life. As far as autism is a lifelong disease, we expected to identify the main symptoms at different periods of development, included adulthood.

## Results

### Prenatal and postnatal administration of VPA decreased the level of social novelty on PND30

**Figure 1:**
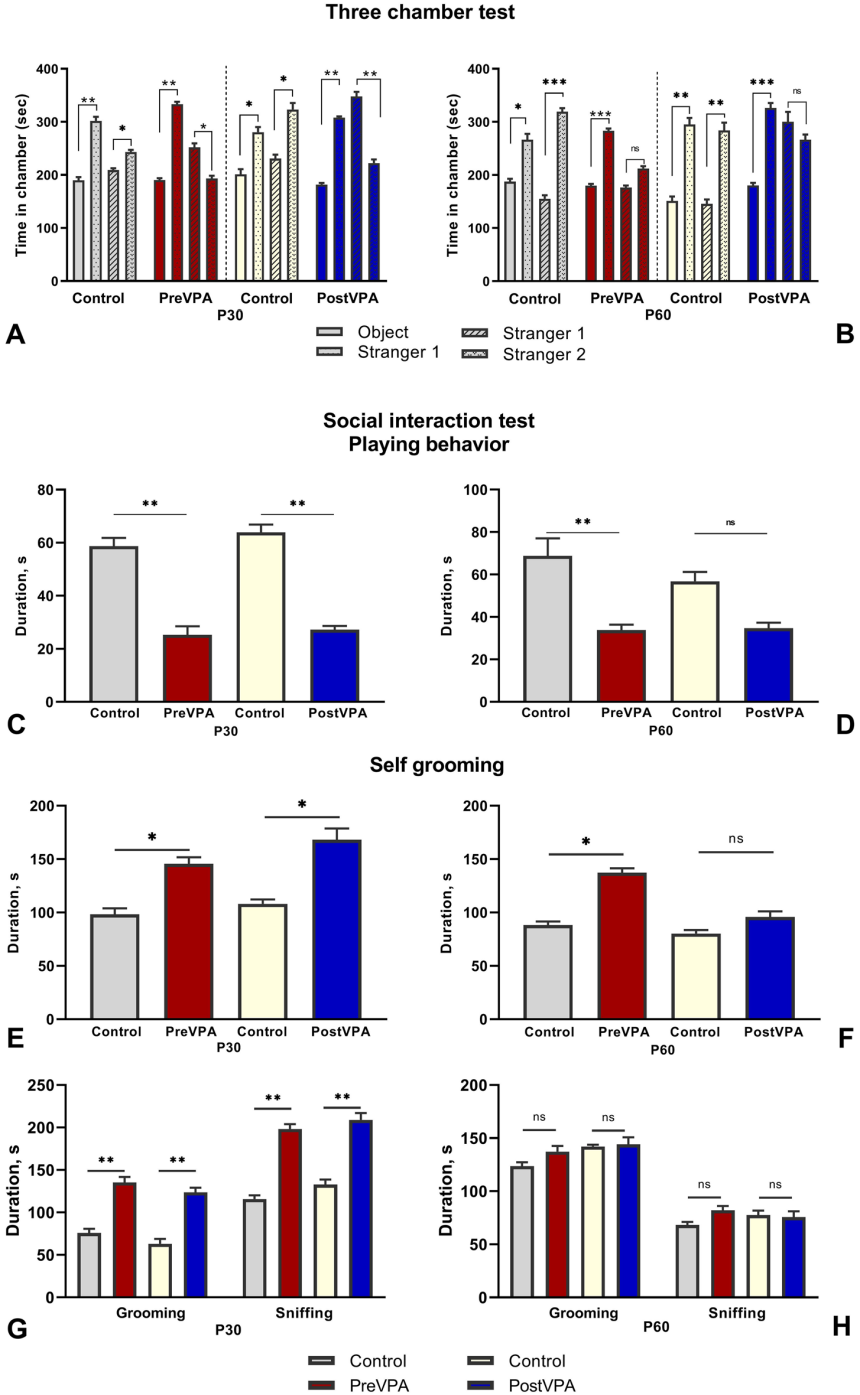
Behavioral alterations associated with VPA treatment. (**A**,**B**) Graphs illustrate time spent in each chamber with Stranger1 and empty cup (object) during the first session, with Stranger1 and Stranger2 during the second session. (**C**,**D**) Graphs illustrate the total duration of pinning and pouncing during the 10 min. (**E**,**F**) Graphs illustrate the total duration of self-grooming (repetitive behavior) during the 10 min. (**G**,**H**) Graphs illustrate the total duration of grooming and sniffing (non-playing behavior) during the 10 min. On the left and right sides of the figure are shown results of the tests carried out on PND30 and PND60 respectively. Data are represented as the mean ± SEM. Statistical significance is indicated as follows: ***p < 0.001, **p < 0.01, *p < 0.05, ns -non-significant.

The three-chamber test is used in rodent models of CNS disorders to access the general sociability and interest in social novelty. Rodents normally prefer to spend time with other rodents and investigate a novel stranger rather than a familiar one. Based on these behavioral features of rodents, the three-chamber test was chosen to identify social deficit and/or social novelty. Our results indicated normal sociability and preference for social novelty in control group on P30 and P60. Rats spent significantly more time in chambers with Stranger 1 and Stranger 2, respectively during the first and second sessions of the test on both of the tested ages (times is sec [mean ± SEM] for control at P30: object 189.9 ± 5.9, stranger 1 301.7 ± 7.9, *p* < 0.01, stranger 1 209.3 ± 3.2, stranger 2 243.6 ± 4.1, *p* < 0.05; control at P60 object 187.4 ± 5.5, stranger 1 266.5 ± 10.9, *p* < 0.05, stranger 1 155.2 ± 6.7, stranger 2 319 ± 6.9, *p* < 0.01,) (Fig. 1A,B). The prenatal VPA-treated rats exhibited normal sociability level on P30 (object 190.2 ± 3.5, stranger 1 333.7 ± 4.6, *p* < 0.001) and P60 (object 179.8 ± 3.3, Stranger 1 283.2 ± 4.3, *p* < 0.001), but they had decreased preference for social novelty on P30 (stranger 1 251.8 ± 7.8, stranger 2 193 ± 5.5, *p* < 0.05), and no significant changes on P60 (Fig. 1A,B).

Results of the second control group indicated substantial degree of sociability at P30 (201 ± 9.7, stranger 1 280.4 ± 9.7, *p* < 0.01) and at P60 (object 151 ± 8.4, stranger 1 295.1 ± 12.5, *p* < 0.01) and preference for social novelty at P30 (stranger 1 230.8 ± 7.3, stranger 2 323 ± 12.6, *p* < 0.05) and at P60 (stranger 1 145.4 ± 8.3, stranger 2 283 ± 14.7, *p* < 0.01) (Fig. 1A,B). The postnatal VPA-treated rats demonstrated normal sociability level at P30 (object 181.8 ± 3.1, stranger 1 307.8 ± 2.5, *p* < 0.01) and at 60 (object 191 ± 4.3, stranger 1 326.2 ± 9.4, *p* < 0.001). In contrast, postnatally VPA-treated rats preferred the chamber with Stranger 1, which indicated a decreased social novelty level at P30 (stranger 1 347.9 ± 8.7, stranger 2 222 ± 7, *p* < 0.01) (Fig. 1A). There were no significant differences at P60, but nevertheless animals preferred chamber with Stranger 1 (stranger 1 300.3 ± 18.5, stranger 2 266.6 ± 9.7) (Fig. 1B).

Interestingly, in three-chamber social approach task VPA-treated rats did not demonstrate social interaction deficits either in adolescence or in adulthood. As the sniffing time was not measured, we supposed that the time spent in lateral chambers is a rather indirect and inaccurate parameter, for sociability assessment, and does not illustrate the level of reciprocal social interaction. Moreover, time spent in chambers may be explained by animals’ hyperactivity, which was also proved in open field test.

### Prenatal and postnatal administrations of VPA altered the social activity and repetitive behavior

Social interaction test is a common method to assess the social impairment criterion relevant to ASD. This test provides the most detailed insights into reciprocal social interaction between pairs of rats placed together. Parameters routinely evaluated include nose to nose sniffing, following, grooming, self-grooming, pinning and pouncing. Playful interactions are most frequent and pronounced in rats between postnatal days 29–34, therefore this period was chosen for first test. Test session lasted 10 min providing sufficient time for habituation and familiarization with each other.

Passive playing behavior was indicated in the prenatal VPA-treated group in comparison with control group at P30 (time in sec [mean ± SEM], control 58.7 ± 3.1, VPA 25.3 ± 3.2, *p* < 0.01) and P60 (control 68.8 ± 8.2, VPA 33.8 ± 2.6, *p* < 0.01) (Fig. 1C,D). The duration of grooming in prenatally VPA-treated group was increased at P30 (control 75.7 ± 5.1, VPA 135.3 ± 6.5, *p* < 0.01) and was normalized at P60 (Fig. 1G,H). Sniffing was also increased in the prenatal VPA group at P30 (control 115.7 ± 4.3, VPA 198.2 ± 5.6, *p* < 0.01) and was normalized at P60 (Fig. 1G,H). The latency of self-grooming was increased on P30 (control 98.3 ± 5.6, VPA 145.5 ± 6.3, *p* < 0.05) and remained unchanged on P60 (control 88.3 ± 3.2, VPA 137.3 ± 4.1) (Fig. 1E,F).

The postnatal VPA-treated group showed a significant decrease in playing behavior at P30 *vs.* the control group (control 63.9 ± 2.9, VPA 27.2 ± 1.4, *p* < 0.01), there were no significant changes at P60 (Fig. 1C,D). Indicators of non-playing behavior were increased in the postnatal VPA-treated rats at P30, such as duration of sniffing (control 132.8 ± 5.8, VPA 208.8 ± 8.1, *p* < 0.01) and grooming (control 63 ± 5.8, VPA 123.6 ± 5.4, *p* < 0.01), whereas there were non-significant differences at P60 (sniffing control 75.5 ± 5.6, VPA 77.5 ± 4.3; grooming, control 142 ± 1.7, VPA 144 ± 6.7) (Fig. 1G,H). Duration of self-grooming was much longer in postnatal VPA-treated group compared to control at P30 (control 108.1 ± 4.1, VPA 168.2 ± 8.8, *p* < 0.05), at P60 no significant differences were detected between the groups (Fig. 1E,F).

### Negative geotaxis

Negative geotaxis test was used as one of the early behavioral tests to motor development (reflexes) and vestibular function.

**Figure 2:**
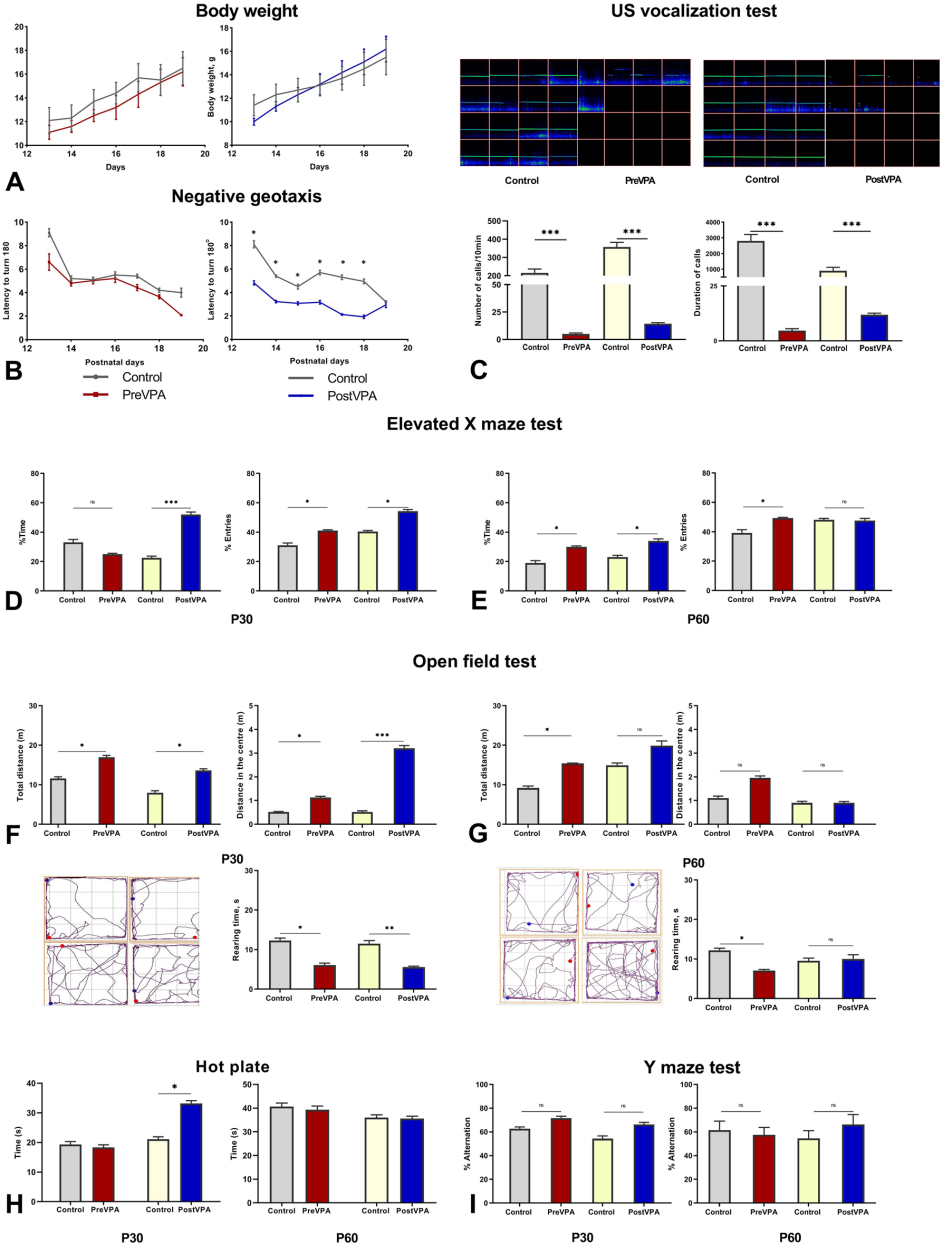
Behavioral alterations associated with VPA treatment. (**A**) Changes in body weight during the PND 13–19 after VPA prenatal and postnatal administration. (**B**) Impairments of ability to turn 180° when pups placed a head down position during the postnatal 13–19 days after VPA prenatal and postnatal administration. (**C**) Exemplary spectrograms of 50 kHz USVs emitted after isolation from mother on PND14, the intensity of the color represents the relative amount of USV with the corresponding frequency and duration respectively. Graphs illustrate duration and number of calls in VPA treated groups vs. Control (ultrasonic emissions were recorded by SPEC’T software, version 3, http://binaryacoustictech.com/batpages_files/spectr.htm, and analyzed by SCAN’R software, version 1.0, http://binaryacoustictech.com/batpages_files/scanr.htm). (**I**) Graphs illustrate the percentage of alternations during the 5 min tested on PND 30 and PND60, the left and right panels respectively. (**D**, **E**) Graphs illustrate (1) ratio of time spent in open arms to total time spent in the maze; (2) ratio of entries’ number into open arms to total entries’ number, tested on PND 30 and PND60, the left and right panels respectively. (**F**, **G**) Graphs illustrate the total distance travelled during the test and distance travelled in central zone, total duration of rearing activity tested on PND30 and PND60, the left and right panels respectively. In the lower left corners are rat's trajectory plots created by Any-maze behavioral tracking software, Stoelting Co. (**H**) Graphs illustrate the duration of response to the thermal stimulus (50 ± 0.5 °C) tested on PND 30 and PND60, the left and right panels. Data are represented as the mean ± SEM. Statistical significance is indicated as follows: ***p < 0.001, **p < 0.01, *p < 0.05, ns -non significant.

There were no significant changes in negative geotaxis test in the prenatal VPA-treated group compared with the control during the all tested days (P13-19). Interestingly, the postnatal VPA-treated group needed less time for completing a 180° turn when placed a head down position (Control group 8.1 ± 0.3, 5.4 ± 0.1,4.5 ± 0.2, 5.7 ± 0.2, 5.3 ± 0.2, 5.0 ± 0.2, 3.2 ± 0.1, Post-VPA group 4.8 ± 0.2, 3.2 ± 0.1, 3.1 ± 0.1, 3.2 ± 0.2, 2.1 ± 0.1, 1.9 ± 0.1, 2.9 ± 0.2, P13-19 days) (Fig. 2B).

The gain in body weight gaining and eye opening days also were examined to assess physical growth and development. There were no significant changes between the tested groups (Fig. 2A).

### Prenatal and postnatal administration of VPA decreased number and duration of calls

Our results showed that prenatal VPA-treated pups emitted significantly fewer calls than the control group (control 216.2 ± 20.9, control 5.0 ± 0.9, *p* < 0.01) and that the duration of the calls was also decreased (control 3129 ± 431.8 ms, VPA 12.1 ± 0.9 ms, *p* < 0.01) (Fig. 2C). Similar to prenatally VPA- treated pups, a decreased number (control 275.5 ± 35.2, VPA 15.5 ± 1.2, *p* < 0.01) and duration of ultrasonic emissions (control 3129 ± 431.8 ms, VPA 12.1 ± 0.9 m, *p* < 0.01) was recorded in postnatal VPA-treated rats (Fig. 2C).

### Prenatal and postnatal administrations of VPA increased locomotor activity and decreased exploratory activity

Open field test is commonly used test to assess total locomotor activity, exploring behavior and anxiety related behavior. Naturally rodents exhibit tendency to explore new environment, typically are more active and remain longer in peripheral zone of apparatus. Distance travelled in peripheral zone avoiding open area indicates level of anxiety, which may be interpreted by thigmotaxis, tendency to remain close to vertical surface.

Prenatal VPA-treated rats travelled more distance in open field than control rats at P30 (control 11.6 ± 0.4, VPA 16.95 ± 0.5, *p* < 0.05), P60 (control 9.3 ± 0.5, VPA 15.4 ± 0.12, *p* < 0.01) (Fig. 2F,G). Distance travelled in the center was also more in VPA-treated group than in control group at P30 (control 0.41 ± 0.05, VPA 1.13 ± 0.05, *p* < 0.05), P60 (control1.1 ± 0.6, VPA 2 ± 1.3, *p* > 0.05) (Fig. 2F,G). In the prenatal VPA-treated group total rearing time was decreased at P30 (control 12.3 ± 0.97, VPA 6.8 ± 0.49, *p* < 0.05), P60 (control 13.2 ± 0.53, VPA 7.07 ± 0.42, *p* < 0.05) (Fig. 2F,G). Self-grooming time was measured as an indicator of repetitive behavior and on both of the time periods it was significantly increased in the prenatal VPA-treated group at P30 (control 9.43 ± 0.85, VPA 18.97 ± 1.36, *p* < 0.05), P60 (control 9.34 ± 0.57, VPA 22.64 ± 1.75, *p* < 0.05).

The total travelled distance in the postnatal VPA-treated group was significantly longer than in control group at P30 (control 11.56 ± 0.37, VPA 16.96 ± 0.47, *p* < 0.05), distance travelled in the center was also significantly more (control 0.52 ± 0.02, VPA 3.21 ± 0.11, *p* < 0.001). Rearing time was significantly decreased in the postnatal VPA-treated group, that indicates low level of exploratory activity (control 11.5 ± 0.7, VPA 5.6 ± 0.2, *p* < 0.01). Additionally, grooming time was significantly increased in the VPA group (control 8.35 ± 0.7, VPA 21.2 ± 1.7, *p* < 0.05). Rats exhibited the same tendency of hyperactivity and low exploratory, but significant differences between the groups were not found at P60 (Fig. 2F,G).

### Prenatal and postnatal administrations of VPA decreased the level of anxiety

Elevated plus maze test is another behavioral method, which helps to assess anxiety-related behavior and is frequently used with the open field test. Similar to open field test rats avoid the open arms, thus the number of entries into open arms and time spend are the most important variables determined in this test.

The prenatal VPA-treated rats showed less anxious behavior during the both tested ages. The number of entries into the open arms was significantly increased in the prenatal VPA-treated group *vs*. control at P30 (control 31.1 ± 1.5, VPA 41.0 ± 0.6, *p* < 0.05) and at P60 (control 39.2 ± 2.2, VPA 49.3 ± 0.5, *p* < 0.05) (Fig. 2D,E). Time spent in open arms was significantly increased in the prenatal VPA-treated group only at P60 (control 23 ± 1.2, VPA 34 ± 1.4, *p* < 0.05) (Fig. 2E).

Both time spent in open arms (control 22.5 ± 1.2, VPA 52 ± 1.7, *p* < 0.001) and the number of entries into the open arms (control 40.4 ± 0.8, VPA 54 ± 1.2, *p* < 0.05) were significantly increased in the postnatal VPA-treated group at P30 (Fig. 2D). Time spent in open arms was increased in the postnatal VPA-treated group compared to control at P60 (control 23 ± 1.2, VPA34 ± 1.4, *p* < 0.05), but there were no significant differences in number of entries (Fig. 2E).

### Prenatal and postnatal administration of VPA does not alter spatial memory

Spatial memory was assessed by the spontaneous alternations in the Y-maze. No significant differences were observed in spontaneous alternation indicating normal working and spatial memory in VPA treated rats in comparison with control group (Fig. 2I).

### Postnatal administration of VPA altered heat nociceptive threshold

Thermal stimulus response was examined to assess VPA effect on rats’ nociception. Test results showed that the postnatal VPA-treated rats significantly delayed the first licking of hind paw at P30 (control 21.1 ± 2.4, VPA 33.2 ± 4.1, *p* < 0.01), that indicate decreased pain sensitivity. There were no significant differences between the groups at P60. Prenatally treated rats did not exhibit significant differences on both time periods (Fig. 2H).

### Prenatal administration of VPA changed the structural characteristics of neurons in PFC and amygdala

**Figure 3:**
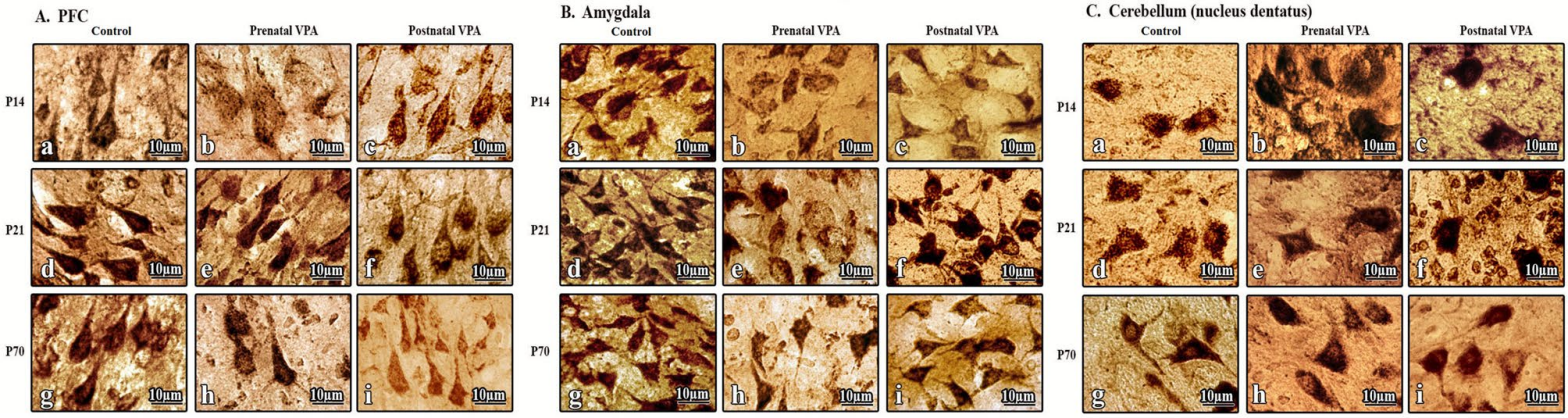
Morphological changes in brain target structures after prenatal and postnatal VPA administration compared to control group. (**A**) After the prenatal administration of VPA on PND 14(b), PND21(e), PND70(h) swollen pyramidal neurons with central chromatolysis and weak staining were found in prefrontal cortex. After the postnatal administration of VPA on PND 14(c), PND21(f), PND70(i) normal pyramidal neurons with centrally located nucleus and long dendrites were found. (**B**) After the prenatal administration of VPA on PND 14(b), PND21(e) swollen pyramidal neurons with central chromatolysis and weak staining were found in amygdala, on PND70(h) structural changes are not pronounced. After the postnatal administration of VPA on PND 14(c), PND21(f), PND70(i) normal pyramidal neurons with centrally located nucleus and long dendrites were found. (**C**) No morphological changes were observed on tested days in cerebellum. Magnification: × 1000 (**A**–**C**).

In prenatal VPA-treated rats morphological changes were detected on all tested time periods. In prefrontal cortex swollen pyramidal neurons with central chromatolysis, weak staining and unclear bound aries were detected at P14. The morphological structure of cells was normalized at P21 and P70 (Fig. 3A). However, there were some cells with central chromatolysis in outer and inner layers of prefrontal cortex. Significant lesions were observed in amygdala on both early and later stages of life. Morphological changes were more pronounced in comparison with the control group at P14 and P21. Neuronal cells became larger and round in shape with reduced and areactive dendrites. In swollen cells nuclei had eccentric location and central chromatolysis was observed (Fig. 3B). Purkinje cells in cerebellum of the prenatal VPA-treated rats mostly retained their normal structure at all tested periods. While there were some Purkinje cells with shortened dendrites and granule cells with reduced sizes. Only several cells of cerebellar nuclei had abnormal morphology with shortened dendrites and swollen nuclei. Also pronounced glial reaction was detected (Fig. 3C).

Results of morphological study showed no significant structural changes in different brain regions after postnatal administration of VPA. Healthy neurons with the normal shape, size and centrally located bright nucleus were detected in all observed brain regions.

### Electrophysiological study

**Figure 4:**
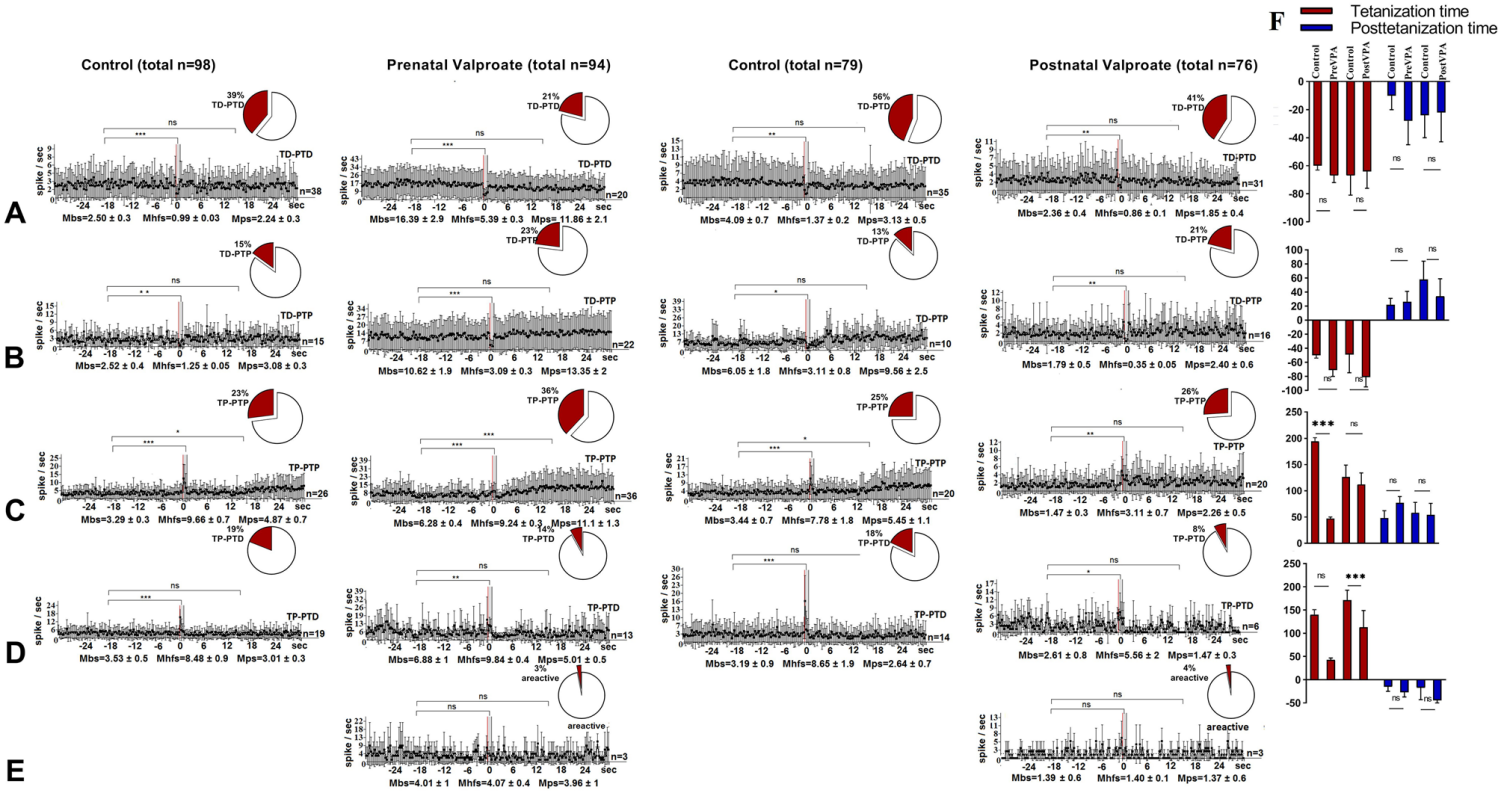
Electrophysiological changes in form of time histograms of peri-stimulus average spike frequency in neurons of prefrontal cortexto high-frequency stimulation of hippocampus after prenatal and postnatal VPA administration. The time histograms of peri-stimulus average spike frequency in neurons of prefrontal cortex are illustrated in the figure. The mean peri-stimulus frequency diagrams built on the basis of pre-stimulus and post-stimulus manifestations of spike activity of single PFC neurons to high-frequency stimulation of hippocampus in a real time 30 s before stimulation (Mbs), 30 s post-stimulation (Mps) and during high-frequency stimulation (Mhfs), exhibiting the specified types of response: (**A**) TD-PTD, (**B**) TD-PTP, (**C**) TP-PTP, (**D**) TP-PTD, and areactivity (**E**) in the VPA treated groups. The data are presented as mean ± SD; n = number of neurons. The statistical significance from baseline (Mbs) was estimated according to the unpaired Student's *t-test*, ***p < 0.001, **p < 0.01, *p < 0.05, ns - non significant. (**F**) The bar diagrams show the average % change relative to the baseline (Mbs, zero level) in responses of tetanization time (red) and post-tetanization time (blue) in control vs. VPA treated groups in the population of neurons with a given type of response. Statistical significance was calculated using Fisher’s exact test, *p < 0.05.

In mPFC during HFS of the hippocampus in Control and prenatal VPA-treated groups 98 and 94 single neurons were recorded (Fig. 4). In the Control 38 neurons out of 98 (38.8%) showed TD-PTD responses during HFS of the hippocampus. The share of such responses in the VPA group decreased non-significantly to 21.3% (20 neurons out of 94) (Fisher's exact test). In these neurons, the frequency of peristimulus spike flow was higher in the VPA group compared to the Control group, both before stimulation Mbs = 16.39 ± 2.9 vs Mbs = 2.5 ± 0.3 spike/sec) and during HFS (Mhfs = 5.39 ± 0.3 vs Mhfs = 0.99 ± 0.03 spike/sec) and poststimulus time (Mps = 11.86 ± 2.1 vs Mps = 2.24 ± 0.3 spike/sec) (Fig. 4A). In both groups, TD was significant (*p* < 0.001, t-Student). In the Control 26 neurons out of 98 (26.5%) showed TP-PTP responses during HFS of the hippocampus. The share of such responses in VPA group increased non-significantly to 38.3% (36 neurons out of 94) (*p* > 0.05, Fisher's exact test) (Fig. 4C). In VPA group the frequency of pre- and poststimulus spike flow of these neurons was higher versus the Control group (6.28 ± 0.4 and 11.1 ± 1.3 vs 3.29 ± 0.3 and 4.87 ± 0.7 spike/sec). At the same time in both groups TP and PTP were significant (*p* < 0.05, Student's t-test). In the VPA group changes in the percentage of TD-PTP and TP-PTD responses in mPFC neurons during HFS of the hippocampus were non-significant (*p* > 0.05, Fisher's exact test), whereas in both groups TP and TD were significant (*p* < 0.05, Student's t test) (Fig. 4B,D). As in populations of neurons mentioned above, in these neurons the frequency of the peristimulus spike flow (Mbs, Mhfs, Mps) was also higher in the VPA group versus Control group.

In Control and postnatal VPA-treated groups 79 and 76 single neurons were recorded in the mPFC during HFS of the hippocampus, respectively. Percentage changes for certain types of responses in the VPA group versus the Control group had the same tendency as for those in prenatally VPA-treated group. In the VPA and Control groups, as in the prenatal VPA-group, in mPFC neurons exhibiting TD-PTD and TP-PTP responses to HFS of the hippocampus, the frequency of spikes during HFS (Mhfs ± SEM) and poststimulus time (Mps ± SEM) was significantly changed (*p* < 0.05, Student's t-test), except posttetanic potentiation in the VPA group (Fig. 4A,C). In both of the groups mPFC neurons exhibited TD-PTP and TP-PTD responses during HFS of the hippocampus, as in the prenatal VPA-treated group, changes in the spike frequency to HFS were significant (*p* < 0.05, Student's t-test). In the postnatal VPA-treated group 3 neurons (4% of the total number of recorded neurons) did not respond to HFS, that was almost consistent with 3.2% of nonreactive neurons in the prenatal VPA-treated group (Fig. 4E). Thus, the frequency of peristimulus spiking (Mbs, Mhfs, Mps) in all recorded neurons was decreased in the VPA group versus the Control, unlike the results of the prenatal VPA- treated group. Percentage change of spike flow mean frequency was also estimated during HFS and after stimulation relative to its basic (prestimulus background) frequency for neurons in particular populations to compare the significance of changes in the prenatal VPA-treated group vs. Control and the postnatal VPA-treated group vs. Control (Fig. 4F). Significant changes were detected in mPFC neurons exhibiting TP-PTP and TP-PTD responses after HFS of hippocampus of the prenatal VPA- treated rats. So, the percentage of TP expression compared to the background spike frequency is 126% in the Control group and it was not significantly reduced to 112% in postnatal VPA-treated group in neurons exhibiting TP-PTP. The same tendency of reduction was detected in prenatally VPA treated group (194% vs 47%, *p* < 0.001, t-test). In population of neurons exhibited TP-PTD, significantly reduced TP was observed in the prenatal VPA-treated group vs. control (43% vs. 140%, *p* < 0.001, *Student's t-test*). There was not significant reduction of TP in the postnatal VPA-treated group vs. control group (113% vs. 171%, *p* > 0.05, *Student's t-test*).

**Figure 5:**
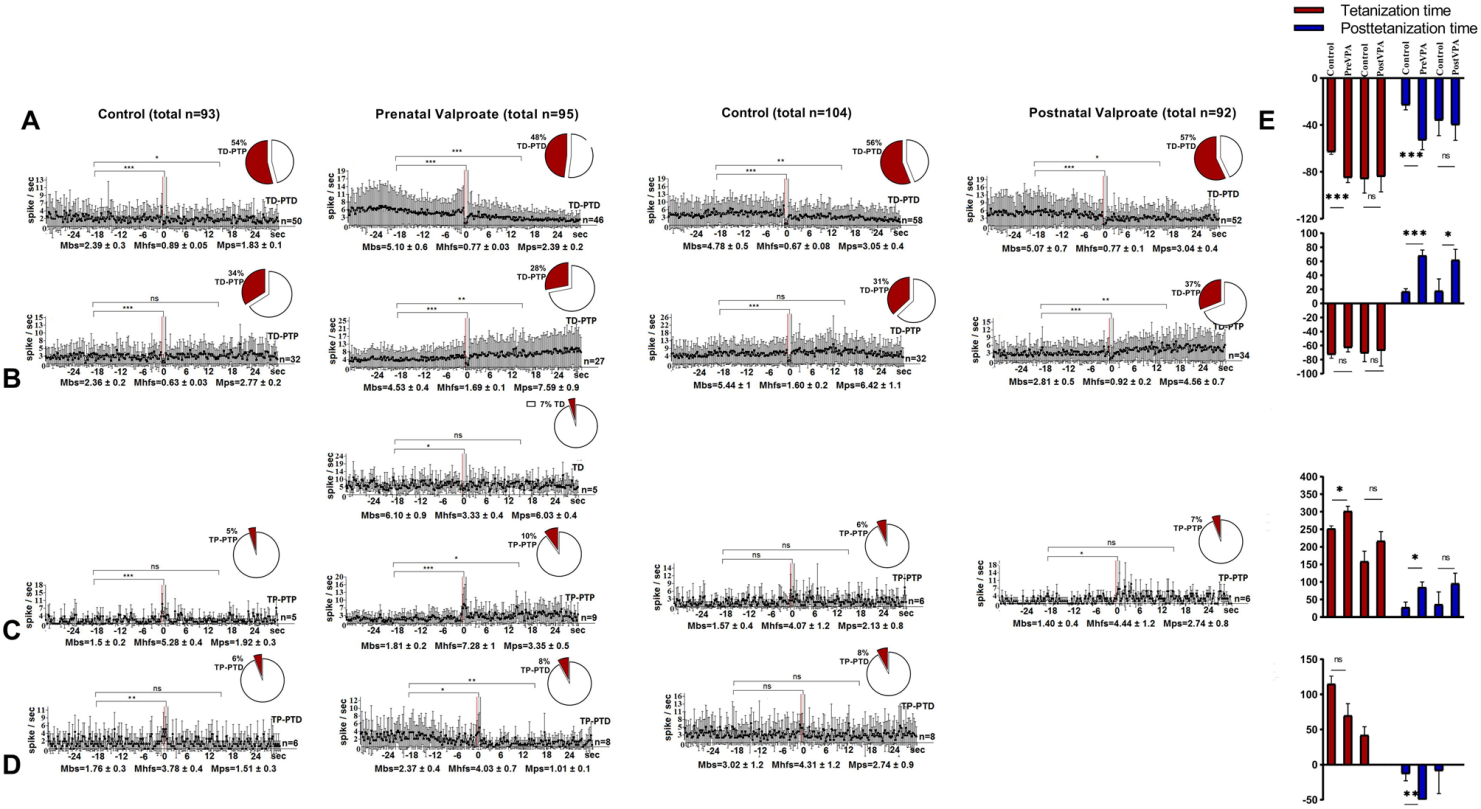
Electrophysiological changes in form of time histograms of peri-stimulus average spike frequency in neurons of prefrontal cortexto high-frequency stimulation of nucleus dentatusafter prenatal and postnatal VPA administration. The time histograms of peri-stimulus average spike frequency in neurons of prefrontal cortex are illustrated in the figure. The mean peri-stimulus frequency diagrams built on the basis of pre-stimulus and post-stimulus manifestations of spike activity of single PFC neurons to high-frequency stimulation of nucleus dentatus of the cerebellum in a real time 30 s before stimulation (Mbs), 30 s post-stimulation (Mps) and during high-frequency stimulation (Mhfs), exhibiting the specified types of response: (**A**) TD-PTD, (**B**) TD-PTP, (**C**) TP-PTP, (**D**) TP-PTD, TD only in prenatally VPA treated group. The data are presented as mean ± SD; n = number of neurons. The statistical significance from baseline (Mbs) was estimated according to the unpaired Student's *t-test*, ***p < 0.001, **p < 0.01, *p < 0.05, ns -non significant. (**E**) The bar diagrams show the average % change relative to the baseline (Mbs, zero level) in responses of tetanization time (red) and post-tetanization time (blue) in control vs. VPA treated groups in the population of neurons with a given type of response. Statistical significance was calculated using Fisher’s exact test, *p < 0.05.

In mPFCof the Control group 50 neurons from 93 (53.8%) showed TD-PTD responses during HFS of nucleus dentatus of the cerebellum (Fig. 5A). The share of such responses in the prenatal VPA-treated group was not significantly reduced to 48.4% (46 neurons out of 95) (*p* > 0.05 Fisher). Generally, there were no significant changes in the percentage ratios of TP-PTP, TD-PTP, TP-PTD responses between the groups (*p* > 0.05 Fisher) (Fig. 5C,B,D), it should be noted that the VPA-treated group had a higher level of frequency pre- (Mbs ± SEM) and post-stimulus (Mps = SEM Mps ± SEM) spike flow. In the VPA group 5.3% of recorded neurons showed only TD response. In both of the groups decreased mean frequency during HFS (TD), as well as increased mean frequency during HFS (TP) were significant compared to the background (prestimulus) frequency (*p* < 0.05, Student's t-test), whereas the poststimulus increase in spike flow (PTP) was significant only in the VPA group.

In mPFC of the postnatal VPA-treated group compared to the Control changes in percentage shares of TD-PTD, TD-PTP, TP-PTP responses during HFS of nucleus dentatus of the cerebellum had the same tendency (non-significant increase or decrease) as in the prenatal VPA-treated group, except TP-PTD (7.7%, *p* < 0.01 Fisher) (Fig. 5D), which was recorded only in the postnatal Control group. However, changes of spike frequencies during TP and PTD were not significant (*p* > 0.05, t-Student). Generally, in the Control group a significant reduction of spike frequency only during TD (0.67 ± 0.08 vs 4.8 ± 0.5 and 1.6 ± 0.2 vs 5.4 ± 1 spike/sec, *p* < 0.001, Student's t-test) and PTD (3.05 ± 0.4 vs 4.8 ± 0.5 spike/sec, *p* < 0.01, Student's t-test) was recorded, while TP-PTP responses were not significant. In the VPA group significantly increased spike frequency during TP (4.4 ± 1.2 vs 1.4 ± 0.4 spike/sec, *p* < 0.05 Student's t-test) and PTP (4.56 ± 0.7 vs 2.8 ± 0.5 spike/sec, *p* < 0.01 Student's t-test) was recorded. At the same time, comparing the percentage changes of the mean frequency with its basic (background) frequency in the VPA group vs. Control group (Fig. 5E) revealed significant changes only with respect to PTP in neurons showing TD-PTP (62 ± 15% vs. 18 ± 17%, *p* < 0.05 Student's t-test). The same indicator revealed significant changes in TD (− 85 ± 4% vs. − 63 ± 2%, *p* < 0.001 t-Student) and PTD (− 53 ± 8% vs. − 23 ± 4%, *p* < 0.001 Student's t-test), TP (302 ± 14% vs. 252 ± 8%, *p* < 0.05 t-test) and PTP (85 ± 15% vs. 28 ± 15%, *p* < 0.05 Student's t-test) in the prenatal VPA-treated group compared to the Control group.

**Figure 6:**
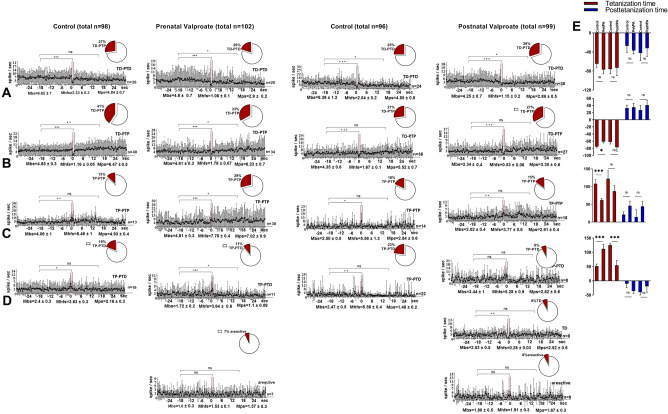
Electrophysiological changes in form of time histograms of peri-stimulus average spike frequency in neurons of amygdala to high-frequency stimulation of hippocampus after prenatal and postnatal VPA administration. The time histograms of peri-stimulus average spike frequency in neurons of amygdala are illustrated in the figure. The mean peri-stimulus frequency diagrams built on the basis of pre-stimulus and post-stimulus manifestations of spike activity of single amygdala neurons to high-frequency stimulation of hippocampus in a real time 30 s before stimulation (Mbs), 30 s post-stimulation (Mps) and during high-frequency stimulation (Mhfs), exhibiting the specified types of response: (**A**) TD-PTD, (**B**) TD-PTP, (**C**) TP-PTP, (**D**) TP-PTD, and nonreactivity in the VPA treated groups. The data are presented as mean ± SD; n = number of neurons. The statistical significance from baseline (Mbs) was estimated acording to the unpaired Student's *t-test*, ***p < 0.001, **p < 0.01, *p < 0.05, ns -non significant. (**E**)The bar diagrams show the average % change relative to the baseline (Mbs, zero level) in responses of tetanization time (red) and post-tetanization time (blue) in control vs. VPA treated groups in the population of neurons with a given type of response. Statistical significance was calculated using Fisher’s exact test, * *p* < 0.05.

98 single neurons were recorded in Amygdala during HFS of the hippocampus in the Control group and 102 neurons in the prenatal VPA-treated group, 7of them (6.8%) were nonreactive (areactive) (Fig. 6). In the Control group 26 neurons out of 98 (26.5%) exhibited TD-PTD responses (Fig. 6A). The share of same responses in the VPA group decreased not significantly to 19.6% (20 neurons out of 102) (*p* > 0.05 Fisher). The percentage share of TD-PTP and TP-PTD responses have also decreased not significantly (Fig. 6B,D), while TP-PTP responses have increased to 29.4% vs. 13.2% in Control group (*p* < 0.05 Fisher) (Fig. 6C). In both of the groups TD and TP responses were significant in all populations, while PTD and PTP were significant in the VPA group, except PTP of neurons showing TD-PTP responses in the Control group (6.47 ± 0.5 vs. 4.85 ± 0.3 spike/sec, *p* < 0.01, Student's t-test).

There was not significant decrease or increase in the percentage shares for TD-PTD, TD-PTP, TP-PTP responses in the postnatal VPA-treated group versus the Control group. In the VPA group nonreactive units (8 neurons out of 99) and neurons showing only TD (8 neurons out of 99) were recorded. A reduction in share ratio of TP-PTD responses was significant (8% vs 23%, *p* < 0.01, Fisher). Comparison of percentage changes of mean frequency with its basic frequency in the prenatal VPA-treated group vs. Control groups (Fig. 6E) revealed a significant decrease in TP (62 ± 5% vs. 108 ± 12%, *p* < 0.0001, Student's t-test) in a population of neurons showing TP-PTP and a significant increase in TP (112 ± 16% vs. 51 ± 8%, *p* = 0.0007 t-Student) in a population of neurons showing TP-PTD. In the postnatal VPA-treated group the same indices decreased significantly only in relation to TP (54 ± 17% vs. 125 ± 7%, *p* < 0.0001 Student's t-test) in neurons showing TP-PTD (Fig. 6E). In Amygdala of the prenatal VPA-treated group a percentage share of excitatory type of responses increased and posttetanic PTD and PTP were significant, TP was increased and decreased significantly. At the same time TD is not significant. The percentage share ratios in the VPA group have changed towards an increased nonreactivity and inhibition, and TP expression was decreased.

## Discussion

**Figure 7:**
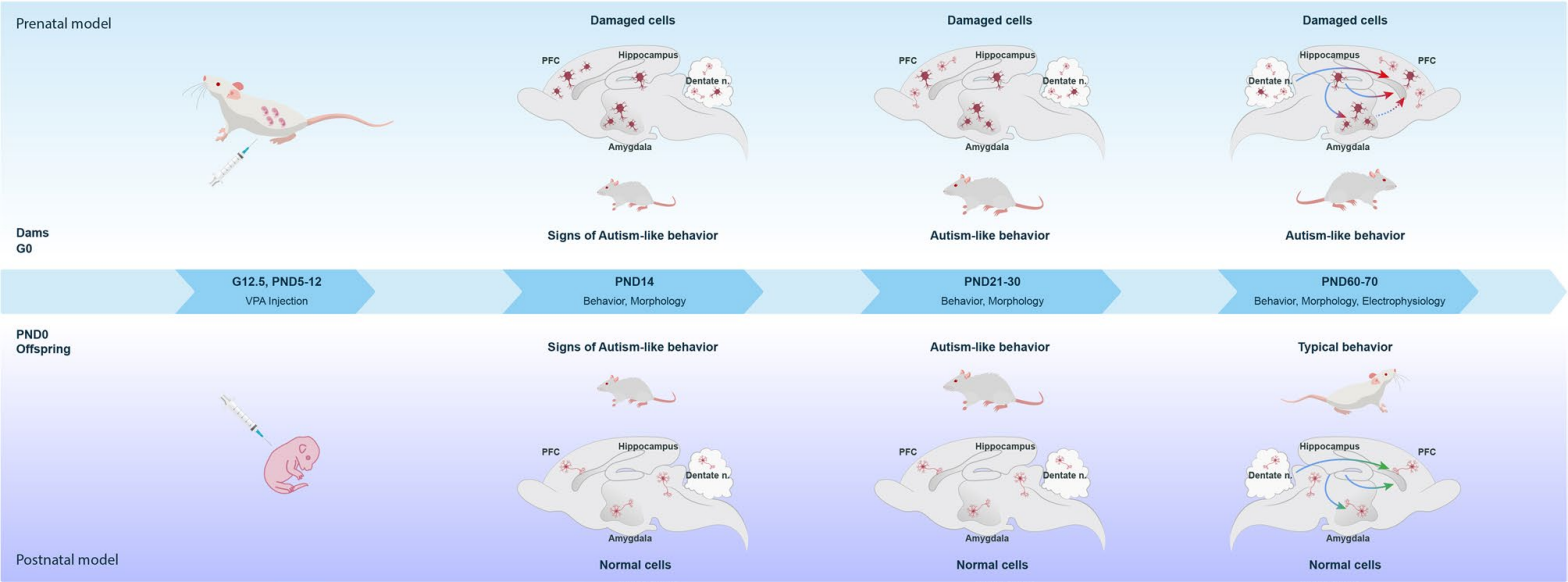
Graphical illustration of behavioral, morphological and electrophysiological changes in pre- and postnatal VPA-induced ASD models in different time periods. In prenatal model increasing ASD-like behavior associated with cellular damage of PFC, amygdala, hippocampus and dentate n. of cerebellum neurons (*damaged cells are in red*) on PND 14, PND (21–30), and PND 60–70 is shown (*upper panel*). Red arrows trace functional alterations between structures analyzed by electrophysiological studies on PND 70. In postnatal model ASD-like behavioral changes associated with normal cellar structure of PFC, amygdala, hippocampus and dentate n. of cerebellum neurons, as well as typical behavior on PND 60–70 are shown. Green arrows trace functional connections between structures analyzed by electrophysiological studies on PND 70 (*bottom panel*).

Results of the current study indicate that both prenatal and postnatal administration of valproic acid have potential role in ASD development. Behavioral alterations associated with ASD^37–39^ were found on early stages of postnatal development (P30). For instance, both prenatally and postnatally VPA treated rats demonstrated decreased latency to playing behavior and preference for social novelty, as well as increased repetitive behavior combined with low exploratory activity. In addition, VPA treated rats showed locomotor hyperactivity and decreased anxiety. After prenatal administration of valproic acid in prefrontal cortex, amygdala and cerebellum cells with abnormal morphology were found on P14 and P21. Interestingly morphological changes in target for autism brain regions were not observed in postnatally treated rats on P14 and P21. These data suggest that valproic acid has a mild effect on brain early postnatal development and does not lead to the structural abnormalities, but possibly disturbs signaling in neural circuits involved in tested behaviors (Fig. 7).

On late stages of development (P70) behavioral alterations remained only in prenatally treated rats. This also indicates the severity of prenatal model in comparison with postnatal one, which exhibits reversible manner of damage. Furthermore, electrophysiological studies confirmed existence of several disconnections between hippocampus, prefrontal cortex and amygdala (Fig. 7). Particularly, an increased frequency of peristimulus spikes were recorded (Mbs, Mhfs, Mps) in all mPFC neurons (both hippocampal and cerebellum HFS) in prenatally VPA treated rats and a contrary trend of low frequency in postnatally VPA treated group versus control. Thus, our results of increased peristimulus frequency in all populations of tested neurons supported the data reported by Ijima et al.^40^ and Rinaldi et al.^33^, which evidence about epigenetic modifications elicited by VPA results in hyperactivity and hyperplasticity in the PFC. Moreover, among the all recorded cells the percentage distribution of PFC neurons exhibited PT-PTP response to hippocampal stimulation was 36% in prenatally VPA treated group, while in control group 39% of cells exhibit PD-PTD. Several studies suggested that activation of pyramidal neurons in the mPFC that project to subcortical areas impaired normal social behavior^41,42^ at the same time hippocampal projections to GABA neurons in mPFC can drive feed forward inhibition onto pyramidal neurons^43^. Recent clinical studies indicated PV + GABA neuron defects in postmortem human brain tissue of patients with autism^44^. Additionally, highfrequency of spikes may be result of increased local connectivity. Several studies indicated increased long term potentiation due to an up-regulation of NMDAR of pyramidal cells of PFC prelimbic region in early adolescence^33,45^. However, recent studies indicated activation of compensatory homeostatic mechanisms, which lead to normalization of synaptic NMDARs from adolescence into adulthood^46^. Previously, the VPA-induced model revealed an increase in the NMDA synaptic currents^47^. The hyper-plasticity in the valproic acid animal model of autism was linked to a massive over-expression of NMDA receptors and might support memory and learning capabilities^47^. It was proposed that deficits in autism such as sociability, attention, multi-tasking and repetitive behavior are consequences of a hyperfunctional prefrontal cortex^47^. Autonomous columns in the prefrontal cortex may excessively lock attention onto specific tasks, which could provide an alternative explanation for the deficits in multi-tasking, specialised interests, behavioural inflexibility or repetitive tendencies, which was observed in our behavioral studies. A recent study suggests that enhanced NMDAR functions in adolescent VPA mice might be compensated by homeostatic events at later developmental stages^48^. Since electrophysiological abnormalities (biophysical parameters) are developmental stage-dependent^48^, it seems that both an increase and decrease of excitatory responses to HFS in amygdala (respectively, in a population of neurons with TP-PTP and TP-PTD), are associated with the fact that register was performed on PD70-80. Our data on unreliable changes in the background activity and inhibitory manifestations correspond to the data of Martin and Manzoni^46^. Our data on unreliable changes of the background activity and inhibitory manifestations correspond to the data of Martin and Manzoni^46^, which find that adult VPA exposed rats show normal level of spontaneous activity and long-term depression. In some time, both NMDAR mediated currents and LTP are lower in adult VPA rats^46^. The presence of mentioned mechanism may explain the absence of synaptic activity in postnatally VPA treated group. Since PFC forming synapses with both amygdala and hippocampus, we consider that observed behavioral alterations also might be associated with disconnection in amygdala-PFC pathway. Infralimbic (IL) and prelimbic (PL) areas of the PFC, which regulate fear extinction and expression processes, receive inputs from amygdala and hippocampus^43^. Evidence supporting this idea includes that stimulation of IL generates extinction like effects, while activities in PL correlates with increased fear behavior. Our results of behavioral tests indicated less anxious behavior in prenatally VPA treated group on P70, withal decreased social behavior. Summarizing the data we hypothesize that it might be loss of PL stimulation by/due to amygdala "silence". Our morphological studies detected swollen neuronal cells with reduced and areactive dendrites mostly in amygdala among of all studied structures on P70. Lesions found in amygdala of VPA treated rats were also positively correlated with social interaction and communication deficit. A number of postmortem studies on autistic patients indicated reduced number of cells in amygdala^49^, decreased neuronal cells sizes^50^.Furthermore, TD and areactivity responses were recorded in amygdala after hippocampal stimulation, which may indicate disconnection between hippocampus and amygdala. Decreased share of TP-PTD and a weak expression of TP also confirm the altered signaling, which correlates with abnormal social behavior. In line with hypotheses that amygdala hyperactivity underlies anxiety^51,52^, in vivo photoactivation of basolateral amygdala–vCA1 synapses significantly increases anxiety-related behaviors, while photoinhibition produces robust anxiolytic effects^53^. As far as hippocampal cells response was not recorded after amygdala stimulation the existence of Hip-amygdala disconnection is not clear. However, received results mostly evidence that in PFC-Amygdala-Hip triad the problem predominantly lies in amygdala.

Increased inhibitory response in PFC after the stimulation of cerebellar nucleus in prenatally VPA treated group may evidence disconnection of glutamatergic or dopaminergic projections to the PFC. This idea may be supported by the number of studies showed disconnection in the autistic brain as a result of developmental cerebellar neuropathology and particularly the loss of Purkinje cells^54–56^. Neuronal circuits originated in cerebellar cortex Purkinje cells project to dentate nucleus, then project to medial PFC via two pathways. The first one involves cerebello-ventral tegmental-cortical indirect circuit, while the second one cerebello-thalamocortical circuit^56^. Some damaged Purkinje cells identified in VPA treated rats may result in loss of cerebellar output leading to a cascade of events which result in the repetitive and hyperactive behavior.

Based on the arguments given above, the severe alterations of synaptic responses, as well as the number of structurally modified cells were more significant in prenatally VPA treated group than in postnatally VPA treated group (Fig. 7). Both of these data confirm the stable and irreversible up to the tested period behavioral changes in prenatally VPA treated group. Observed behavioral alterations after postnatal administration of valproic acid proved susceptibility of premature brain to the early stressors including drugs and environmental toxicants.

## Conclusion

**Figure 8:**
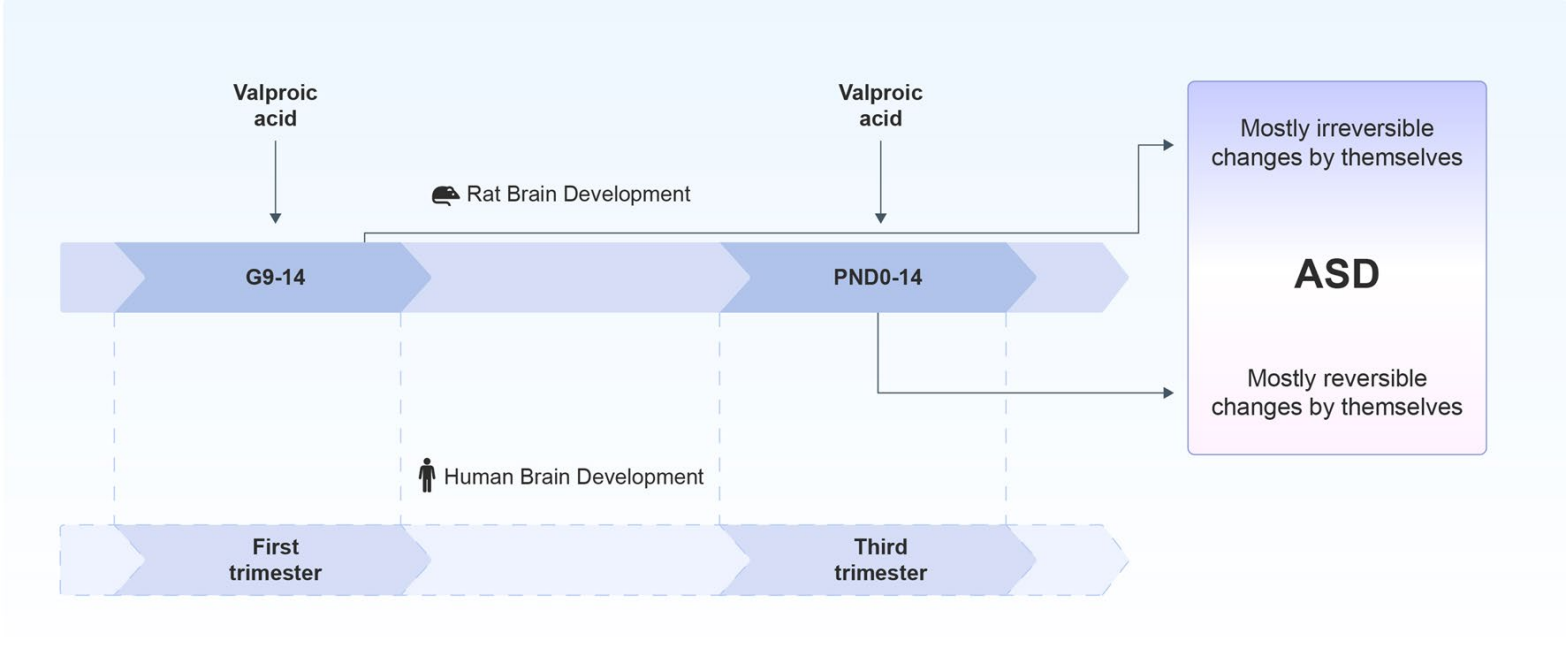
Possible ASD phenotypes modeled in rats by exposure to valproic acid in different periods of brain development in rodents and humans. Exposure to valproic acid on gestation day 12.5 of pregnant rat which corresponds to the 1st trimester of human pregnancy lead to manifestation of stable and irreversible ASD phenotype. While administration of valproic acid during the first two weeks of postnatal life, which corresponds to the 3rd trimester of human pregnancy, later is manifested with reversible ASD like symptoms. Mostly they appear at early adolescence and normalized up to adulthood by themselves.

Thus, our results confirm previously described studies that a single prenatal exposure to VPA results lifelong behavioral impairments similar to core symptoms of autism. In comparison to well-known and widely used embryonic model postnatal administration of VPA also lead to the autistic features in adolescent rats which showed tendency to normalization in adulthood. We found pronounced structural changes in the brain target regions of prenatally VPA-treated groups, and an absence of abnormalities in postnatally VPA-treated groups, which confirmed the different severity of VPA across different stages of brain development. (Fig. 8). The differential responsiveness to prenatal and postnatal VPA injections observed in our study might be explained by temporal differences of brain regions development process. Thus, postnatal model may be useful for the assessment of damaging effect of VPA on neurobehavioral development that parallels the clinical signs of autism. Moreover, investigations of compensatory homeostatic mechanisms activated after VPA administration and directed to eliminate the defects in postnatal brain, may elucidate strategies to improve the course of disease.

## Methods

### Animals

Experiments were performed on adult 12–14 months of age male and female Sprague–Dawley rats which were housed together overnight to obtain pregnant rats for prenatal model, as well as pups of 5 days for postnatal model, young rats in prepubertal adolescence (30–40 days) and adulthood (60–70 days) periods of age. All animals were purchased from university vivarium and kept under conditions—12-h light/dark cycle, with controlled temperature (22 ± 2 °C) and free access to food and water. All experiments and methods were performed in accordance with relevant guidelines and regulations. The experimental protocol corresponded to the conditions of the European Communities Council Directive (86/609/EEC) and was approved by the Ethics committee of Yerevan State Medical University after Mkhitar Heratsi. The study was carried out in compliance with the ARRIVE guidelines for animals.

### Experimental design

Two variants of VPA-induced autism models were used—prenatal and postnatal. The main behavioral features (social interaction, repetitive behavior, anxiety, locomotor activity, thermal nociception), which are associated with ASD core and co-occurring symptoms were tested on postnatal 30–40 (P30) and 60–70 (P60) days. For behavioral studies 8–12 rats were used in each experimental group. Neuroanatomical lesion of autism target structures (prefrontal cortex, amygdale, cerebellum) was observed on14th (P14), 21st (P21) and 70th (P70) days of postnatal development respectively. Electrophysiological studies in specific neural circuits, such as hippocampus—mPFC, nucleus dentatus of cerebellum—mPFC, hippocampus—basolateral amygdala were carried out on P70. Morphological and electrophysiological studies were carried out on 3 rats from each experimental group respectively. In sum, all rats were divided into four groups (Control and Prenatal model, Control and Postnatal model) by 18–20 in each.

### Modeling of ASD

#### VPA prenatal administration

Male and female rats were housed together overnight. Females’ pregnancy was detected by spermatozoa found in vaginal smear after overnight mating, which was recorded as day 0 of gestation. Pregnant females received intraperitoneal single injection of sodium valproate 500 mg/kg (Valproic acid, sodium salt, Abcam 120745) dissolved in 0.9% saline on embryonic 12.5 day (E12.5). Control females received saline solution on E12.5.

#### VPA postnatal administration

Day of birth was recorded as postnatal day 0 (P0), pups remained with dams until the weaning day P23. Pups received intraperitoneal injections of sodium valproate 200 mg/kg (Valproic acid, sodium salt, Abcam 120745) dissolved in 0.9%saline, and control pups received saline on postnatal days 5–12 (P5–12). Pups’ development was assessed by measuring of body weight, detecting eye opening day and evaluation of negative geotaxis reflex.

### Behavioral studies

The battery of different behavioral test was performed on P30–40 and P60–70 periods each day between 12:00–6:00 pm in the same sequence. From 8 to 12 animals were used for each behavioral test. The same animals were used in all behavioral tests.

#### Negative geotaxis

Negative geotropism was measured on P13-19 by placing the pup facing down along 45° incline. Latency to turn 180° was recorded. Each pup was tested 3 times and the average time was used in analysis.

#### Ultrasonic vocalization test

Rat pups are completely dependent on their mother for surviving and they emit USVs in response to isolation. Pups isolation-induced vocalization at frequencies between 30–65 kHz usually peaking during the first two weeks of postnatal life and decreasing until weaning. Pups remaining silent during the first few minutes after separation from the mother and habituate, therefore duration of the test is also important aspect to consider.

Taking into consideration all mentioned above, pups were tested immediately after valproate postnatal injections on 14th postnatal day and the test lasted 10 min. Pups were isolated from their mothers and placed into polypropylene cage (30 × 20 × 12 cm) with the controlled temperature (22 ± 2 °C) and covered with bedding material. Ultrasonic vocalization was recorded by microphone which was lowered into the cage to a height 10 cm above the bedding surface. Ultrasonic emissions were recorded by SPEC’T software, version 3.0 and analyzed by SCAN’R software, version 1.0. Total number of calls over the 10 min and their duration were measured.

#### Three chambers test

Sociability and social novelty test apparatus was a rectangular box (60 × 40x25cm, LxWxH) divided into three chambers with holes which allow free movement throughout the box. Test was done by three phases. During the first phase (habituation) tested rat was placed in the box for 2 min. After this phase two cups with small holes allowed olfactory contact between rats were placed in two peripheral chambers. Unfamiliar naive rat (Stranger 1) with the same age and sex as tested one was placed under the cup, the second cup remained empty. Test duration was 10 min. Immediately, after the first phase a new unfamiliar naive rat (stranger 2) was placed under the second cup and tested animal remained in a box additional 10 min. Apparatus was cleaned by 70% ethanol after each animal testing. Time spent in each chamber during the two phases was recorded. Animal sociability was evaluated by preference for the cup with rat versus empty one. Social novelty was evaluated by preference for the Stranger 2 versus Stranger 1. All recorded videos were analyzed manually.

#### Social interaction test

Animals were tested in Plexiglas box (60 × 40 × 25 cm) and paired with unfamiliar partners (control vs. control, VPA treated vs. VPA treated) of the same sex and weight (± 5 g). Before the test on the same day all animals were isolated for 3.5 h, after that simultaneously placed in cage for 10 min. Cage was cleaned by 70% ethanol after each animal testing. Tests were video recorded and analyzed by observer manually. The total duration of playful social interaction (pinning, pouncing) and unrelated to social play behavior (following, sniffing, grooming) was scored for each pair. Pinning described as one of the rats is lying on the dorsal surface while the other rat standing over it, pouncing—one rat soliciting another one were chosen as specific parameters of rodent’s playing behavior. Latency to sniffing and grooming of any part of the body were measured as parameters of unrelated to play behavior, latency to self-grooming was measured as an indicator of repetitive behavior.

#### Open field test

Locomotor activity of rats was tested in an open field apparatus (1 × 1 m). Rats were placed in the center of open filed apparatus and allowed to move freely for 5 min. Apparatus was cleaned by 70% ethanol after each animal testing. All recorded videos were analyzed by AnyMaze software (ANY-maze behavioral tracking software, Stoelting Co). Open field was divided into center and peripheral zones, total distance and distance travelled in the center zone were quantified. Exploratory activity in a novel environment was assessed by recording of rearing time.

#### Elevated plus maze test

Anxiety-related behavior was examined in the standard elevated 50 cm above the floor plus maze. Rats were placed in the center of the maze, facing an open arm. Apparatus was cleaned by 70% ethanol after each animal testing. Test was recorded during 5 min and analyzed with recording of the following two parameters: (1) ratio of time spent in open arms to total time spent in the maze; (2) ratio of entries’ number into open arms to total entries’ number. All recorded videos were analyzed manually.

#### Y maze

The Y maze spontaneous alternation test has been used to assess repetitive behavior and spatial working memory. Rats were placed in the clean three arms apparatus facing to the same arm for 5 min. Tests were video recorded and analyzed by AnyMaze software (ANY-maze behavioral tracking software, Stoelting Co). After each trial apparatus was cleaned by 70% ethanol. The number of alternated entries was scored and divided by the total number of entries.

#### Hot plate

Sensitivity to thermal nociception was determined by a measuring the latency of response to the thermal stimulus (50 ± 0.5 °C) during 60 s on a hot plane analgesia meter. As a response signal was taken the first licking of hind paw.

### Electrophysiological studies

The area-specific activity statistics such as spike-timing and firing rates in the prefrontal cortex and the amygdala of rats was studies upon high-frequency stimulation (HFS) of the hippocampus or the cerebellum. Electrophysiological studies were carried out on P70-80 rats. The used anesthetic was Urethan (1.1 g/kg, i/p), animals were immobilized with 1% dithylinum (25 mg/kg, i/p), position was the stereotaxic frame and artificial ventilation was used. After fixing on the brain, the strict atlas coordinates of the study structure (both for recording and stimulation) are marked and a 2 mm diameter is drilled with a machine. Using a macro screw, the stimulation electrode is then lowered dorsoventrally to the required depth into this opening. The stimulatory bipolar cylinder electrode was inserted following the stereotaxic coordinates in the hippocampus (AP-2.5–3; L ± 2.5–3; DV + 2–2.5 mm) and nucleus dentatus of cerebellum (AP-11–11.3; L ± 2.4–3.4; DV + 6.5–6.8 mm)^57^. The recording electrode we fabricate from the borosilicate glass capillaries (2 mm outer diameter, 0.5 mm wall thickness, Hilgenberg, Mansfeld, Germany) (tip diameter 1–2 μm, resistance, 1.5–2.5 MΩ) and fill with 2 M NaCl. The recording electrode is also lowered dorsoventrally to the required atlas depth using a macro screw, but then lowered using a micro screw with a baseline oil—in at least 10 micron increments—so that no single neuron encountered on a given track is damaged. Recording electrode repeatedly plunged into the basolateral amygdala and mPFC (the coordinates were AP-4.2; L ± 3.4–4.2; DV + 8–9 mm and AP + 3.2; L ± 0.6; DV + 3.6 respectively)^57^ to record spike activity flow of a single neuron. Voltage signals were amplified and filtered. These procedures ensured the adequacy of the brain area for each rat, and thus the adequacy of the recorded neuronal units. In some experiments, the pipette solution was supplemented with 0.2% biocytin (Sigma Aldrich) to allow staining of cellular morphology after the experiment. High frequency stimulation (HFS) (100 Hz during 1 s) was achieved using the rectangle charge for 0.05 ms with 0.08–0.10 mA amplitude. The control of the intensity of stimulation for an individual neuron was based on threshold of response. Each subsequent recording from a different neuron in a given track was done with 3–5 min intervals (7–10 min intervals on average if no neuron was encountered). When the high frequency components of the induction paradigm are optimal for STP and a gap in stimulation is introduced the persistence of transient reactions becomes readily apparent^58^. Recording and analysis of the spike activity of single neurons were carried out by the software (SpikeRegistrator, Rg 1 MFC Application, Rg 1 EXE) < sup > 62,63 < /sup >  < sup > 62,63 < /sup > that selects spikes by amplitude discrimination and allows estimation duration of interspike intervals (or spike frequency, spike/sec) in a real time within 30 s before stimulation (pre-stimulusepoch or background activity), 1 s of HFS (tetanization epoch) and 30 s after stimulation (post-stimulus epoch)^59,60^. Our experience has shown that if the threshold stimulation parameters (for hippocampus and cerebellum in this study) are properly chosen to elicit responses in single neurons (of amygdala and prefrontal cortex), the neuron exhibits a fairly stable level of background and post-stimulatory activity. Sussillo et al. (2007) show—through extensive computer simulations—that STP works as a homeostatic mechanism that stabilizes the overall activity level in spite of drastic changes in external inputs and internal circuit properties, while preserving reliable transient responses to signals^61^.

**Figure 9:**
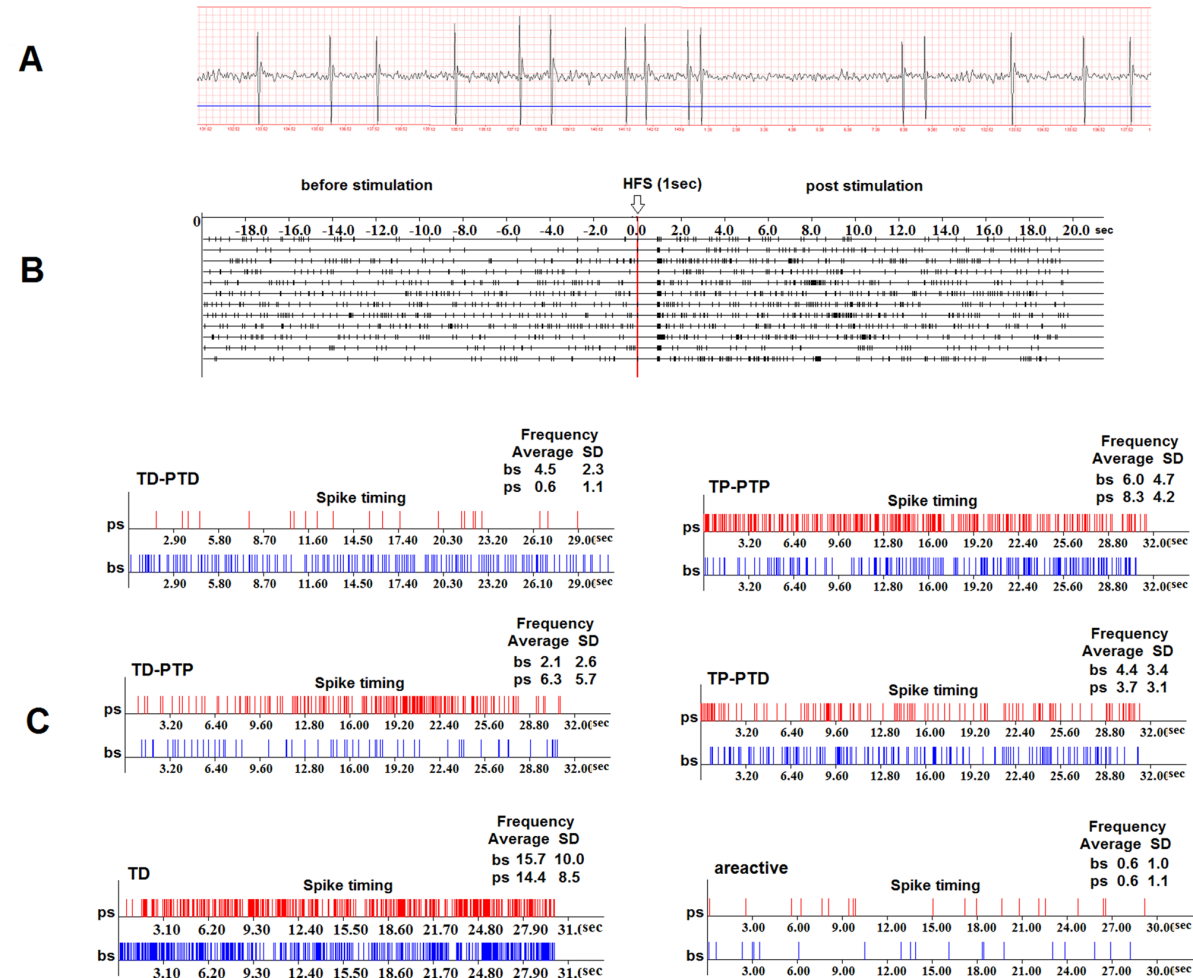
(**A**) In vivo recorded spike flow of single neuron in mPFC. The blue line is amplitude discriminator for making a spike selection. (**B**) The spikes distribution in real time before stimulation (bs), post stimulation (ps) and during high frequency (HFS) stimulation (1 s on ps) for mPFC single neurons specific types of responses. (**C**) Raster of spikes peri-stimulus distribution in real time produced by individual hippocampal neurons (12 trials at time intervals of 5 min). Recording and analysis of the spike activity of single neurons were performed by the software (SpikeRegistrator, Rg 1 MFC Application, Rg 1 EXE).

After recording of neuronal activity in animals of a given group, all single-unit recordings are sorted/arranged by response type. The analysis of spike activity revealed an impulse flow acceleration during HFS (tetanic potentiation, TP) and post-stimulus time (post-tetanic potentiation, PTP), as well as an impulse flow deceleration during HFS (tetanic depression, TD) and post-stimulus time (post-tetanic depression, PTD). The different combinations of responses such as TD-PTD, TD-PTP, TP-PTP were recorded (see Fig. 9). The statistiscal significance of the heterogeneity of interspike intervals (or spike frequency) of the pre- and post-stimulus impulse flow was analyzed by the *Student's t*-*test and* Mann–Whitney U test. Thus, we recorded single-cell spiking short-term dynamics of a large range, which are presynaptically induced by HFS and these results confirm the view, that in a large-size network, STP can greatly enrich the network's dynamical behaviors^62^.

In fact, despite the fact that PTP and PTD are thought to be history-dependent processes, Taufer and Kumar (2021) investigated feed forward networks with excitation and synaptic inhibition, with target dependent STP, and found that this arrangement allows for extra robustness (or system stability) for the output gain (or transmission coefficient)^63^.

In each experimental group the relative contribution of specified type of responses was calculated as a percentage share of the analyzed neurons. The statistical significance percentage share of specified types of responses between Control and Valproate groups was estimated according to the chi square Fisher's exact test. The average peri-event time histogram (PETH) of neurons with uniform responses was constructed on the basis of analysis of peri-stimulus firing rate for given populations. Intra-population variance of frequency change during HFS and post-stimulation time compared to prestimulus background activity was calculated using unpaired *Student's t-test*. The statistical significance of the mean % changes/difference spikes frequency during tetanization time and posttetanization time relative to baseline (Mbs, zero level) in comparative groups Valproate vs Control was estimated according to the unpaired *Student's* t-test in a population of neurons with specified type of responses.

### Morphological study

To visualize and assess the brain cells structure the activity of Ca^2+^dependant acidic phosphatase was identified. This method based on the modification of Nissl staining and Golgi silver impregnation^60^ that allows to identify not only large but also small neural cells. Brains were isolated and fixed on P14, P21 and P70. Brains were fixed in the 5% buffered neutral formalin (contained 0.1 M phosphate buffer (pH 7.4), 0.3% CaCl_2_, 15% sucrose) for 24–48 h at 4C°. The frontal free-flow frozen slices (40–50 µm thick) of prefrontal cortex, amygdale and cerebellum were taken. After the washing in distilled water slices were transferred into the incubation mixture containing 0.4% lead acetate, 1 M acetate buffer (pH 5.6), 2% sodium glycerophosphate for 2–3 h at 37 °C. The slices were thereafter washed in distilled water, transferred into 3% sodium sulfide solution, rewashed in distilled water and embedded into Canada balsam.

### Statistical analysis

The results were statistically assessed by Student's t-test for independent samples, and in the case of nonhomogenicity by Kolmogorow–Smirnow and Mann–Whitney U-tests. Weight gain and ontogeny of negative geotropism were analyzed by ANOVA with the day of testing as a repeated measure followed by LSD post hoc test. P values below 0.05 were considered as statistically significant.

The statistical significance of the heterogeneity of interspike intervals (or spike frequency) of the pre- and post-stimulus impulse flow was analyzed by Student’s *t*-test and Mann–Whitney U test. The average peri-stimulus time histogram (PSTH) of neurons with uniform responses was constructed based on the analysis of peri-stimulus firing rate for given populations. Intra-population variance ratio between time intervals during HFS and post-stimulation compared to baseline was calculated using Student’s *t*-test. The statistical significance between control and VPA-treated groups was estimated according to chi-square and Fisher’s exact tests^62^.

### Ethical approval

The experimental protocol corresponded to the conditions of the European Communities Council Directive (86/609/EEC) and was approved by the Ethics committee of Yerevan State Medical University after Mkhitar Heratsi (Protocol).

## Data Availability

Data can be made available by the corresponding author upon reasonable request.
